# Spatial Distribution of Recurrence and Long-Term Toxicity Following Dose Escalation to the Dominant Intra-Prostatic Nodule for Intermediate–High-Risk Prostate Cancer: Insights from a Phase I/II Study

**DOI:** 10.3390/cancers16112097

**Published:** 2024-05-31

**Authors:** Minna Cloître, Sofian Benkhaled, Sarah Boughdad, Niklaus Schaefer, John O. Prior, Michele Zeverino, Dominik Berthold, Thomas Tawadros, Jean-Yves Meuwly, Paul Martel, Chantal Rohner, Leonie Heym, Frederic Duclos, Véronique Vallet, Massimo Valerio, Jean Bourhis, Fernanda G. Herrera

**Affiliations:** 1 Department of Oncology, Radiation Oncology Service, Centre Hospitalier Universitaire Vaudois, 1005 Lausanne, Switzerland; minna.cloitre@chuv.ch (M.C.); sofian.benkhaled@chuv.ch (S.B.); michele.zeverino@chuv.ch (M.Z.); leonie.heym@chuv.ch (L.H.); frederic.duclos@chuv.ch (F.D.); veronique.vallet@chuv.ch (V.V.); jean.bourhis@chuv.ch (J.B.); 2 Department of Medical Imaging, Nuclear Medicine Service, Centre Hospitalier Universitaire Vaudois, 1005 Lausanne, Switzerland; sarah.boughdad@chuv.ch (S.B.); niklaus.schaefer@chuv.ch (N.S.); john.prior@chuv.ch (J.O.P.); 3 Department of Oncology, Medical Oncology Service, Centre Hospitalier Universitaire Vaudois, 1005 Lausanne, Switzerland; dominik.berthold@chuv.ch; 4 Department of Surgery, Urology Service, Centre Hospitalier Universitaire Vaudois, 1005 Lausanne, Switzerland; thomas.tawadros@hopitalrivierachablais.ch (T.T.); paul.martel@chuv.ch (P.M.); massimo.valerio@hcuge.ch (M.V.); 5 Department of Medical Imaging, Radiology Service, Centre Hospitalier Universitaire Vaudois, 1005 Lausanne, Switzerland; jean-yves.meuwly@chuv.ch (J.-Y.M.); chantal.rohner@chuv.ch (C.R.); 6 Ludwig Cancer Research Center Lausanne, 1005 Lausanne, Switzerland

**Keywords:** pattern of recurrence, prostate cancer, intermediate–high-risk, SBRT, stereotactic treatment, dosimetry analysis

## Abstract

**Simple Summary:**

This reassessment of the findings from our phase I/II study seeks to evaluate the effectiveness of SBRT for treating local intermediate–high-risk prostate cancer in the absence of androgen deprivation therapy due to patient refusal, with a median follow-up of 6.5 years. Our primary focus is on analyzing the treated areas compared to recurrence patterns in the seven patients who experienced local relapse. Additionally, we provide updated data on late toxicities of grade ≥ 2, both genitourinary and gastrointestinal.

**Abstract:**

**Objectives:** We investigated spatial patterns between primary and recurrent tumor sites and assessed long-term toxicity after dose escalation stereotactic body radiation therapy (SBRT) to the dominant intra-prostatic nodule (DIN). **Materials and methods:** In 33 patients with intermediate–high-risk prostate cancer (PCa), doses up to 50 Gy were administered to the DIN. Recurrence sites were determined and compared to the original tumor development sites through multiparametric MRI and ^68^Ga-labeled prostate-specific membrane antigen (PSMA) positron emission tomography/computed tomography (^68^Ga-PSMA-PET/CT) images. Overlap rates, categorized as 75% or higher for full overlap, and 25–74% for partial overlap, were assessed. Long-term toxicity is reported. **Results**: All patients completed treatment, with only one receiving concomitant androgen deprivation therapy (ADT). Recurrences were diagnosed after a median of 33 months (range: 17–76 months), affecting 13 out of 33 patients (39.4%). Intra-prostatic recurrences occurred in 7 patients (21%), with ≥75% overlap in two, a partial overlap in another two, and no overlap in the remaining three patients. Notably, five patients with intra-prostatic recurrences had synchronous bone and/or lymph node metastases, while six patients had isolated bone or lymph node metastasis without intra-prostatic recurrences. Extended follow-up revealed late grade ≥ 2 GU and GI toxicity in 18% (n = 6) and 6% (n = 2) of the patients. **Conclusions**: Among patients with intermediate–high-risk PCa undergoing focal dose-escalated SBRT without ADT, DIN recurrences were infrequent. When present, these recurrences were typically located at the original site or adjacent to the initial tumor. Conversely, relapses beyond the DIN and in extra-prostatic (metastatic) sites were prevalent, underscoring the significance of systemic ADT in managing this patient population. **Advances in knowledge:** Focal dose-escalated prostate SBRT prevented recurrences in the dominant nodule; however, extra-prostatic recurrence sites were frequent.

## 1. Introduction

Prostate cancer (PCa) is the most frequent malignancy in men, with 1.4 million new cases diagnosed each year, and 90% of them have a confined tumor [1].

Radiotherapy (RT) is a well-established curative treatment option. Combining androgen deprivation treatment (ADT) with RT has been shown to improve overall survival (OS) among patients with intermediate- and high-risk PCa [2,3,4]. Several studies have demonstrated that dose-escalated external beam radiation treatment (EBRT) has better biochemical control than standard-dose radiation [5,6], and dose escalation with a brachytherapy boost may be an even better option [7,8,9]. 

Furthermore, since the publication of three randomized controlled trials on hypofractionated radiation therapy, this approach can now be considered a standard of care, even for high-risk patients [10,11,12,13].

As more data support the use of SBRT for localized PCa, several investigators attempted to raise RT dosages to the whole prostate, demonstrating that 45 Gy and 47.5 Gy were safe. However, increasing the whole-prostate dose to 50 Gy resulted in higher levels of severe late toxicity [14,15].

Recurrences after EBRT are typically seen in the dominant intra-prostatic nodule (DIN), which is the largest nodule with the most aggressive biological behavior, and studies of patterns of failure following the standard 78 Gy EBRT have revealed that the DIN was the primary site of tumor recurrence in more than 90% of patients [16,17]. However, there is a limited amount of evidence on the patterns of recurrence after SBRT, particularly patterns of intra-prostatic recurrence after dose-escalated SBRT to the DIN. 

We previously reported a prospective phase I/II study, in which we used SBRT to irradiate the whole prostate gland with tumoricidal doses of 36.25 Gy in five fractions while simultaneously increasing the RT dose to the DIN up to 50 Gy [18]. To achieve this, the protocol mandated the use of a rectal balloon spacer to maximize rectal protection and fiducial markers. In that study, we implemented cutting-edge imaging techniques, such as multiparametric magnetic resonance imaging (mpMRI), which has been shown to improve the sensitivity and specificity in detecting and characterizing high-risk PCa foci [19], as well as ^68^Ga-labeled prostate-specific membrane antigen (PSMA) positron emission tomography/computed tomography (^68^Ga-PSMA-PET/CT) in order to determine the site of recurrence and compare it to the original site of tumor development.

In this reanalysis of our phase I/II study, we looked at the spatial pattern association between primary and recurrent tumor sites after prostate SBRT with dose escalation to the DIN. Importantly, 97% of our patients had intermediate- or high-risk PCa and only one of them received ADT, allowing us to assess the pattern of recurrence of RT administered in the absence of ADT.

## 2. Materials and Methods

This study constitutes a phase I/II dose escalation trial, sanctioned by the Ethics Committee of the Canton Vaud (ClinicalTrials.gov ID: NCT02254746). Written informed consent was obtained from all participants. Between October 2014 and April 2017, we enrolled eligible patients diagnosed with previously untreated prostate cancer (PCa), classified as low-, intermediate-, or high-risk according to the D’Amico risk classification [20]. Patients included had stage T2 to T3 adenocarcinoma of the prostate, with no nodal (N0) or distant metastasis (M0). In the case of PSA ≥ 20 μg/L, and/or T3 tumor and/or Gleason Score ≥ 8, the patient must have undergone a bone scan and a CT scan of chest–abdomen and pelvis. 

All patients were required to have at least one visible nodule on mpMRI. Serum PSA levels had to be <50 μg/L, and the International Prostate Symptom Score (IPSS) had to be ≤15 (alpha blockers permitted). Exclusion criteria included pre-SBRT prostate volume on MRI exceeding 70 cm^3^ or tumors located within 3 mm of the urethra upon mpMRI assessment. 

All patients were counseled in the prostate cancer tumor board in presence of a medical oncologist (DB), a urologist (TT, MV), and a radiation oncologist (FH, JB). ADT was offered to all patients in this context.

Concomitant or adjuvant ADT was allowed, but neoadjuvant ADT was an exclusion criterion.

The primary endpoint of the phase I study was to assess acute (up to 90 days after the first RT fraction) urinary and rectal toxicity. This subsequent analysis provides data on long-term toxicity as well as pattern of recurrence.

### 2.1. Radiotherapy Planning and Delivery

A biodegradable spacer (BioProtect Balloon™ Implant system, BioProtect Ltd., Tzur Igal, Israel) was perineally implanted between the prostate and the rectum under transrectal ultrasound guidance, with the patient under sedative anaesthesia. During the same procedure, four gold anchor fiducial markers (Gold Anchor, Naslund AB, Stockholm, Sweden) were inserted into the prostate, ensuring at least 2 cm of spacing between them to meet the fiducial spacing threshold of at least 1 cm in orthogonal imaging for precise rotational corrections. To satisfy collinearity criteria, all angles formed by at least three fiducials had to exceed 15°. Planning MRI and planning computed tomography (CT) scans were conducted 1 to 7 days following fiducial and balloon insertion. To prevent anatomical changes in the rectum that could affect image fusion, the planning MRI was immediately followed by a planning CT scan. To ensure accurate fusion, both planning MRI and planning CT scans were performed with a uniform slice thickness of 1 mm [21]. 

Following the insertion of rectal spacer/fiducial markers, planning T2-weighted MRI image sets were rigidly fused to planning CT images (fiducials-based registration), omitting the use of a catheter for urethral visualization. This method was employed to ensure contouring accuracy and appropriate visualization of the DIN and organs at risk (OARs). To minimize prostate motion during planning scans and treatment, several precautions were implemented. Patients were advised to adhere to a low-fiber diet starting 5 days before planning scans until treatment completion to reduce intestinal gas. A moderate laxative was administered 48 h prior to planned MRI and CT scans. Enemas were performed if necessary 1 h before planning scans and before each treatment session to minimize rectal volume. Patients were encouraged to drink 200 mL of water 1 h before scans, following complete voiding. Anatomical contours of the prostate, DIN, seminal vesicles, and OARs were delineated by lead investigators (F.H. and J.B.), then reviewed by a panel of board-certified radiation oncologists. Identification of the DIN and urethra as the region of interest on MRI was conducted by a radiologist (J.-Y.M.). The planned target volume was generated by uniformly expanding the prostate by 3 mm (PTVp). The DIN was outlined as the gross tumor volume (GTV) and extended by 3 mm to form a PTVDIN, without employing a clinical target volume around the GTV. The prescribed dose to the PTVp was 36.25 Gy administered in 5 fractions (7.25 Gy per fraction) at the 80% isodose line.

The prescribed dose to the PTV_DIN_ was 45, 47.5, and 50 Gy in 5 fractions, corresponding to the 80% isodose line; hence, the maximum dose point corresponded to 56.25, 59.38, and 62.5 Gy, respectively. To allow gradients for DIN boosting and to maximize PTV_DIN_ doses, dose heterogeneity had no limits. At least 95% of the volume of interest (PTV_DIN_ and PTVp) needed to be covered by >95% of the prescription dose. A treatment fractionation schedule mandated a minimum of 2 days and a maximum of 6 days between fractions, with no more than 2 fractions per week. The overall treatment duration was capped at 26 days.

Dose–volume histogram goals and RT planning details were previously published [22]. Briefly, dose–volume histogram goals for the rectum included a maximum dose of 0.1 cm^3^ < 41 Gy and V25 < 20 (volume receiving 25 Gy < 20 cm^3^). Bladder dose–volume histogram limits required <0.1 cm^3^ to receive <45 Gy, and the bladder median dose was capped at 20 Gy. Urethra dose was restricted to <1 cm^3^ of urethra receiving >39 Gy, with <0.1 cm^3^ not to exceed 41 Gy.

Patients were preferably treated with CyberKnife; however, Tomotherapy (Accuray Inc., Sunnyvale, CA, USA) or VMAT (Elekta Synergy Stockholm, Stockholm, Sweden) were permitted in cases of CyberKnife unavailability. Treatment utilized 6 MV energy, with orthogonal X-ray imaging for image guidance based on fiducial marker positions. In CyberKnife-treated patients, X-ray image shifts from planning CT scans were monitored in real time during each fraction. Tomotherapy or VMAT sessions were interrupted every 10 min to allow for re-scanning and verification of prostate and fiducial marker positions.

To enhance patient comfort, all patients were treated supine with a knee cushion.

### 2.2. Treatment Schema 

The whole prostate received a radiation dose of 36.25 Gy in 5 fractions at 7.25 Gy each. Dose escalation to the DIN followed a traditional 3 + 3 design for the phase I part of the study [23]. Dose-limiting toxicities (DLTs) were defined as grade ≥ 3 gastrointestinal (GI) or genitourinary (GU) toxicity occurring from the first fraction of RT up to 90 days after completing treatment, assessed using the National Cancer Institute Common Terminology Criteria for Adverse Events (NCI-CTCAE, version 4). Patients received an initial DIN dose of 9 Gy per fraction up to 45 Gy. If no DLT occurred in the first cohort, then an additional 3 patients were enrolled at the next dose level (47.5 Gy in 5 fractions of 9.5 Gy). Dose escalation continued until DLT was observed or until the maximum tolerated dose (MTD, 50 Gy in 5 fractions of 10 Gy) was reached without DLT. If DLT occurred in a cohort, then an additional 3 patients were enrolled at that level. If 2 or more patients experienced DLT, then a lower dose level was explored to determine the MTD. Patients could be enrolled simultaneously or sequentially within a cohort. However, dose escalation was not allowed until the last patient in a cohort completed a minimum 90-day follow-up without DLT. Following completion of the phase I trial, an interim analysis was conducted, and the trial was reviewed by an Independent Data Safety Monitoring Board (IDSMB) to determine its continuation to phase II. Patients treated at the MTD or with DLT in phase I were included in the phase II analysis.

### 2.3. Phase I/II Design 

The phase II part of the study used a Simon optimal two-stage design [24] to calculate sample size, using grade ≥ 2 acute GU and GI toxicity occurring during treatment, and up to 90 days after completion of SBRT as the endpoint. We considered a grade ≥ 2 toxicity rate of 10% as acceptable for promising treatment, whereas a rate of 70% or higher was deemed unacceptable. We set a 5% probability of accepting the treatment as acceptable if the true toxicity-free rate was 70% or lower (alpha), and a 20% probability of rejecting the treatment if the true toxicity-free rate was 90% or higher (beta). An interim safety analysis by the IDSMB was conducted after treating the first 6 patients at the recommended phase II dose (RP2D). If 2 or more patients out of 6 developed grade ≥ 2 GU or GI toxicity, then the trial was halted; otherwise, an additional 21 patients were enrolled in the second stage. After treating 27 patients in phase II, the treatment would be considered promising for further study if 5 or fewer patients developed grade ≥ 2 GU or GI toxicity within the initial 3 months of SBRT. All adverse events were graded according to NCI-CTCAE, version 4. Secondary endpoints included late toxicity (occurring >90 days from first fraction), PSA kinetics, and patient-reported outcomes. Baseline and post-treatment assessments at 1, 3, and 6 months included European Organization for Research and Treatment of Cancer QoL Form PR25 (prostate module) and IPSS scores. QoL evaluations were optional after 6 months but collected when feasible. Health-related QoL outcomes were assessed using the European Organization for Research and Treatment of Cancer guidelines [25] scored from 0 to 100. A 10-point difference or more was deemed clinically significant [26]. 

Primary endpoint results (safety) and secondary endpoints including biochemical control and quality of life were recently published elsewhere [22]. 

### 2.4. Determination of Recurrences 

Patients were monitored with PSA measurements and physical examinations every 3 months for the first 2 years, and every 6 months thereafter. Biochemical recurrence was determined using the nadir +2 μg/mL failure definition [27]. Upon confirmation of biochemical recurrence, patients were staged using ^68^Ga-PSMA-PET/CT and prostate mpMRI, which were evaluated by dedicated and experienced nuclear medicine physicians (J.P., S.B., N.S.) and radiologists (J.-Y.M.) in order to determine the exact site of failure. Any focal 68Ga-PSMA-11 uptake above location-specific background levels was considered PSMA-positive. This is the definition according to the largest prospective analysis of PSMA-11 in over 2000 patients [28]. We determine the distance between the DIN and the urethra/rectum and its associated grade ≥ 2 toxicity. Results were considered significative with a *p*-value ≤ 0.05 using Welch’s correction. Analyses were conducted in Prism 7 (GraphPad Software, Inc., La Jolla, CA, USA). 

The ^68^Ga-PSMA-PET/CT image set was fused with the initial planning CT, allowing recurrence locations to be delineated in order to determine the delivered doses to the local recurrent area. Additionally, to corroborate the location of the recurrence, mpMRI was deformably fused with the ^68^Ga-PSMA-PET/CT images and the planning CT. 

The recurrent tumor volume was delineated in the planning CT. 

The prostate was divided into 12 sections (left/right, anterior/posterior, and base/mid-gland/apex), and the seminal vesicles were divided into 2 sections (left/right) [29]. The primary DIN and recurrent tumor were judged separately as present or absent in each section. We computed the overlap rate between the primary DIN and the recurrent tumor. The overlap rate was defined as the number of sections that overlapped between the primary DIN and recurrent tumor divided by the total number of sections of the primary DIN. A recurrent tumor was judged to be in the “same location” if there was an overlap equal to or greater than 75%. A “partial overlap” was defined as an overlap rate between 25 and 74%. A recurrence was considered to be in a “different location” from the DIN if the overlap rate was 24% or less. 

## 3. Results

### 3.1. Pattern of Recurrence

All patients received SBRT to the prostate using 36.25 Gy in five fractions of 7.25 Gy prescribed at the 80% isodose line, with dose escalation to the DIN up to 50 Gy in five fractions of 10 Gy. Despite ADT being offered to the entire patient population, only one patient accepted to be treated with concomitant ADT. 

Table 1 shows the most important patient and tumor characteristics. In total, 23 patients underwent scintigraphy at baseline according to their risk group to confirm the absence of metastasis. Among them, 12 underwent an additional CT scan. Additionally, 2 patients benefited from a ^68^Ga-PSMA-PET/CT. At a median follow-up of 82 months (range: 68–130 months), the PSA failure rate was 39.4% (N = 13). PSA rebounds were found in three patients, two of whom later recurred. In all 13 biochemically recurred patients, ^68^Ga-PSMA-PET/CT and/or mpMRI revealed local or systemic recurrences. Out of the 13 patients that recurred, 69% (N = 9) had high-risk (HR) disease according to the D’Amico risk classification [20] and 30% (N = 4) had intermediate-risk (IR) disease. Notably, 30% (N = 4) of the patients with recurrent disease harbored a T3a/b disease. 

**Figure 1:**
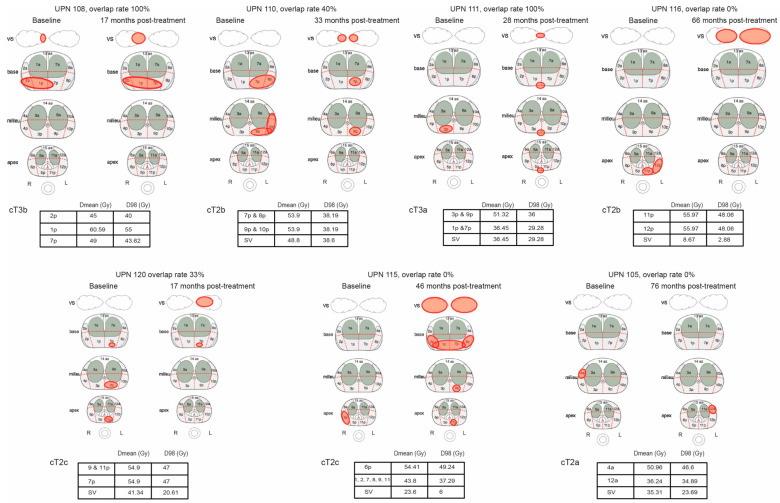
Tumor lesions in the different prostate areas in the patients with local recurrences. Tables show the Dmean and D98% received in each DIN region. Overlapping recurrences were calculated using the formula: number of sections (primary DIN)/number of sections overlapping (between primary DIN and recurrence).

Intra-prostatic recurrences occurred in seven patients (21%), and the median time from diagnosis to local recurrence was 33 months (range: 17–76 months). We computed 100% overlap in two patients, a partial overlap in two others, and no overlap in the remaining three. Recurrent tumors received a median Dmean of 42.57 Gy (range 8.67–60.59 Gy) and D98% of 36.09 Gy (range: 2.88–55 Gy). Interestingly, the seminal vesicles were affected in all recurrent tumors except one. Figure 1 describes the location of the DIN in each individual patient as well as the site of recurrence with their respective overlap rate and Dmean/D98% doses administered. 

**Figure 2:**
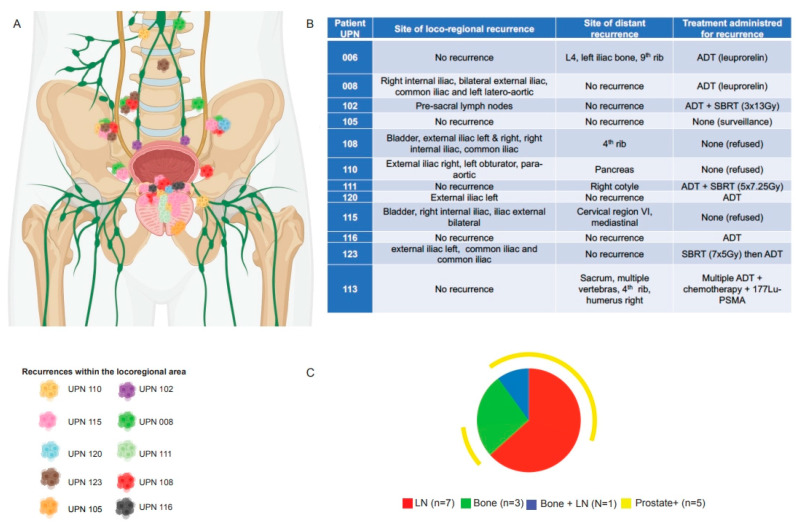
Locoregional recurrences in individual patients. (**A**) Anatomical location of locoregional recurrences; each color represents one patient (UPN, unique patient number). (**B**) The table highlights the specific site of locoregional recurrence for each patient, as well as the site of distant metastases and the appropriate treatment received at recurrence. (**C**) The Spice graphic was generated using Spice software (V6). The graph illustrates the overlap of locoregional and distant metastases: bone metastases are depicted in green, lymph node metastases in red, and both bone and lymph node metastases in blue. The yellow circuit represents prostate recurrences, which did not occur in isolation but in conjunction with locoregional and/or distant metastases.

Notably, five of the seven patients with intra-prostatic recurrences exhibited synchronous metastases: three in the lymph nodes, one in the bone, and one in both. On the other hand, six patients presented with either bone (N = 2) or lymph node (N = 4) metastasis in the absence of an intra-prostatic recurrence (Figure 2). Supplementary Figure S1 details a patient’s case who recurred 28 months after treatment completion. 

Upon recurrence, ADT was recommended to most of the patients and refused by three of them. One patient was put under clinical and radiological follow-up after a board decision. One patient died of metastatic disease.

### 3.2. Safety 

**Figure 3:**
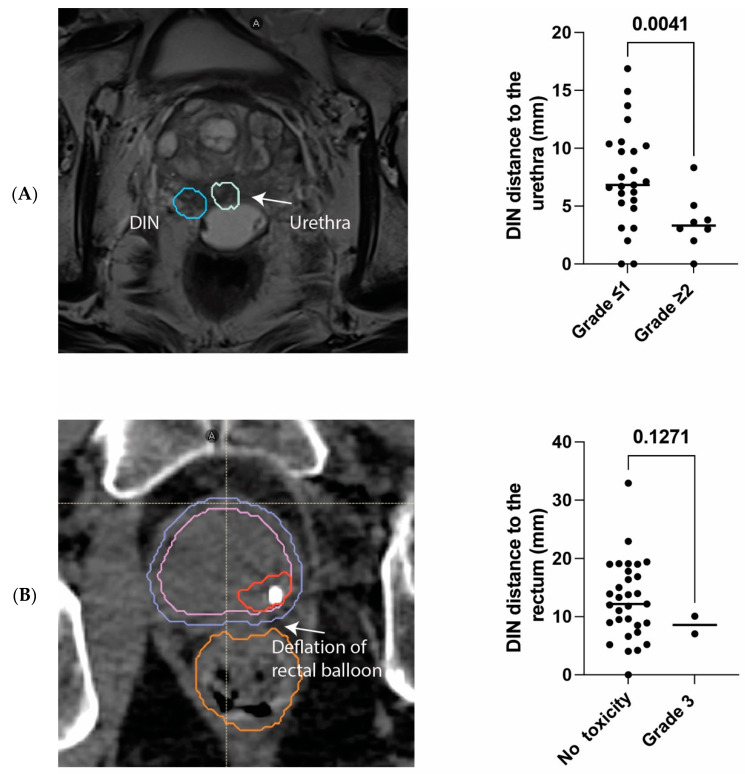
Panel (**A**): Scatter plot of individual patient’s values depicts median distance between DIN and urethra in patients with or without late grade ≥ 2 genitourinary toxicity. Image shows the baseline MRI of a patient with grade 2 late urinary toxicity. Panel (**B**): Scatter plot of individual patient’s values depicts median distance between DIN and rectum in patients with or without grade 3 late gastrointestinal toxicity. Image shows planning CT of a patient with grade 3 late gastrointestinal toxicity (fecal incontinence), demonstrating the deflation of his rectal spacer.

Toxicity appeared at a median of 25 months (range: 9–54 months), and grade 2 and 3 late GU toxicity was present in 18% (N = 6) and 3% (N = 1) of the patients, respectively (Table 2). Specifically, unique patient number (UPN) 113 was admitted to the hospital 4.5 years after treatment completion for urethral stenosis and a urinary infection that necessitated the placement of a temporary catheter. Grade 3 late GI toxicity appeared in two patients (6%) (Table 2). Specifically, UPN 111, who had undergone repeated surgical resections due to hemorrhagic diverticulosis prior to SBRT, had hemorrhagic diverticulosis 51 months after SBRT. Similarly, UPN 110 developed fecal incontinence 6 months after the end of SBRT, which was presumably caused by distal deflation of the rectal spacer (Figure 3). There was no grade 2 late GI toxicity.

In patients with late grade ≥ 2 GU toxicity, the mean distance between the PTV-DIN and the urethra was 3.6 mm compared to 7.5 mm in patients without significant signs of toxicity (*p* < 0.0041) (Figure 3). There was no statistically significant difference in mean prostate volume between patients with and without toxicity (64.5 mL versus 57.3 mL, *p* < 0.29). The distance between the rectum and the DIN was not significantly shorter in patients with late grade 3 GI toxicity (Figure 3). 

## 4. Discussion

In the first five years following treatment, 5 to 15% of PCa patients who had current hypofractionated RT will experience a biochemical recurrence, and the rate of recurrences varies according to the initial disease risk [30]. There has been a noticeable decrease in local failures in recent studies [31] adopting focally dose-escalated radiation schemes, although the prostate and seminal vesicles continue to be the most common initial recurrence sites after conventional RT. 

In this study, which remains small and failed to provide effective ADT treatment due to patient refusal, we found a 39.4% cumulative incidence of recurrence at 6.5 years, with 69% of recurrent patients having a high-risk disease and a failure pattern of distant metastases in most patients. Among those with distant metastases, five patients (45%) also experienced a local recurrence, which was also predicted by an unfavorable cancer pathology at baseline (Figure 1), implying that precise patient selection is necessary for the local treatment to be effective, and that the delivery of ADT is important. Local recurrence was shown to originate within the initial tumor site in two patients, a partial overlap in another two (40 and 33% OR), resulting in a 12% intra-DIN recurrence, and no overlap in three patients (9% extra-DIN), indicating that, although speculative, residual tumors after DIN dose-escalated SBRT expanded to areas adjacent to the original site and/or recurrences occurred in an original microscopic multifocal tumor. According to the FLAME phase III trial [32], which evaluated the benefit of focal boost to the DIN in the context of EBRT and ADT, the biochemical disease-free survival rate was significantly better in the focal-boosted arm (HR 0.45; 95% CI: 0.28–0.71, *p* < 0.001), with only 3% of local intra-prostatic recurrences [31]. Due to the small number of local recurrences compared to the number of distant metastases, our study provides preliminary information for the rationale of adding a focal boost to SBRT. 

In the ASCENDE-RT trial, which compared a DIN boost using brachytherapy to external beam irradiation alone, 95 patients experienced biochemical progression. Based on randomization, the 10-year Kaplan–Meier time to progression (TTP) was 67% for the dose-escalated EBRT (DE-EBRT) arm, compared to 85% for the brachytherapy-boosted arm (*p* < 0.001). Within the National Comprehensive Cancer Network (NCCN) intermediate-risk group, the 10-year Kaplan–Meier TTP was 73% for the DE-EBRT arm versus 90% for the brachytherapy-boosted arm (*p* = 0.02). In the NCCN high-risk group, the 10-year Kaplan–Meier TTP was 64% for the DE-EBRT arm compared to 81% for the brachytherapy-boosted arm (*p* = 0.006) [7,9].

This study experienced significant challenges, including difficulties in treatment positioning and planning; for example, the accuracy of mpMRI and CT planning fusion was achieved by implementing fiducial-marker-guided rigid fusions and a rectal balloon spacer. On the other hand, hot spots located in close proximity to healthy organs (urethra, bladder, and rectum) had to be manipulated in order to respect dose constraints. All of these challenges may raise the possibility of missing the target, especially in those four patients in whom the disease appeared in close proximity to the DIN, which we cannot rule out. Recurrent tumors received a median Dmean of 42.57 Gy (range 8.67–60.59 Gy) and D98% of 36.09 Gy (range: 2.88–55 Gy). The lower range limit (8.67 and 2.88 Gy) corresponded to three patients who recurred in non-irradiated seminal vesicles; two of these patients had large prostate volumes, and in order to limit sigmoid doses, irradiation of the seminal vesicles was omitted in view of the absence of infiltration at baseline. ^68^Ga-PSMA-PET/CT was not available at our institution at the time patients were recruited into the trial; therefore, we relied on mpMRI scans. It is possible that the incorporation ^68^Ga-PSMA-PET/CT would have helped to identify eventual millimetric disease that could have spread around the DIN. Despite these limitations, our results could serve as baseline data addressing the lack of high-level evidence regarding these issues. A recent meta-analysis of 38 prospective SBRT series, including over 2900 patients with intermediate-risk disease, reported a 5-year biochemical recurrence-free rate of 92.1% [33]. These outcomes were not stratified by favorable or unfavorable intermediate-risk groups; nevertheless, the available evidence suggests that SBRT-treated men with unfavorable intermediate-risk PCa have equal outcomes when compared to conventionally fractionated radiation. The recently published HYPO-RT-PC randomized phase III non-inferiority trial demonstrated identical 5-year failure-free survival rates between ultra-hypofractionated and conventionally fractionated radiation therapy. They included 1200 patients, 89% of whom were at intermediate risk [34]. A recent article of completed prospective trials that pooled results for low- and intermediate-risk patients did stratify results into favorable and unfavorable intermediate-risk groups. The 7-year biochemical failure-free survival rate was 93 and 85% for favorable intermediate- and unfavorable intermediate-risk groups, respectively [30]. This excellent outcome is confirmed by another multicentric prospective trial, with 5-year rates of 100 and 93.1%, respectively, for favorable intermediate- and unfavorable intermediate-risk patients [35]. Additionally, the previously mentioned pooled trial results indicate low rates of distant metastatic disease (1.7% and 3.0%, respectively), without prostate cancer mortality. In our own analysis, eleven patients developed metastatic disease, with one patient dying from prostate cancer, and two patients had intra-prostatic recurrence only.

Our higher incidence of metastatic failures is probably linked to the higher proportion of high-risk patients (55% of the trial population) and the lack of ADT in the majority of them. Data supporting the use of SBRT in high-risk prostate cancer are more limited. The previously mentioned meta-analysis included 470 patients with high-risk disease but could not provide an estimate of biochemical freedom from recurrence for this cohort, as many studies did not report estimates based on risk group. Numerous retrospective and single-institution studies have included 7.4% to 65.9% high-risk patients, with variable total doses (32–40 Gy in five fractions) and the use of ADT [36,37,38,39,40,41,42,43]. Additionally, a large National Cancer Database study found no difference in overall survival between SBRT and conventionally fractionated patients when propensity-matched for high-risk subpopulations with Gleason scores of 8+ or PSA >10 µg/L [44]. Despite potential selection bias, the results are promising with a 5-year biochemical failure-free recurrence between 70 and 80% in most series; therefore, our study is aligned with those results. One of the largest series of high-risk patients (n = 52) with a long follow-up (median 60 months) is the Katz et al. study, which reported a 6-year biochemical failure-free survival rate of 69%. This is comparable to the 7-year rate of 68% seen in high-risk patients treated with dose-escalated conventionally fractionated radiation. The HYPO-RT-PC trial included 11% high-risk patients but did not report biochemical outcomes by risk group. Due to the small proportion of high-risk patients, the authors concluded that there is insufficient evidence to support SBRT as a standard of care for high-risk patients, unlike their conclusion for intermediate-risk patients. Furthermore, this trial did not include ADT, which is the standard of care for high-risk patients [34]. A consortium of seven institutional phase II studies (SHARP) investigated the efficacy and toxicity of SBRT in 344 high-risk prostate cancer patients [45]. Most trials within the consortium administered SBRT to the entire prostate, with doses ranging between five fractions of 7 to five fractions of 8 Gy. With a median follow-up of 49.5 months, 72% of patients received ADT (median duration of 9 months), and some patients underwent elective nodal radiotherapy. The 4-year biochemical recurrence-free survival was 81.7% (95% CI, 77.2–86.5%), and the distant metastasis-free survival was 89.1% (95% CI, 85.3–93.1%). The crude incidences of late grade ≥ 3 genitourinary and gastrointestinal toxicity were 2.3% and 0.9%, respectively. 

Due to the rising concerns associated with clinical observations of significant late toxicity in other dose escalation trials [14], we present data with a long-term follow-up focused on treatment safety. Although late grade 3 toxicity remained low (3% GU and 6% GI) in this study, we demonstrated a significant association between the volume of irradiation and the risk of late toxicity in patients treated with dose-escalated SBRT. To place the results of the present series in the appropriate context, we extracted the rates of late severe (i.e., grade 3 or higher) toxic events after treatment reported in other series a with long-term follow-up (Supplementary Table S1) [22,38,42,46,47,48,49,50,51,52,53,54,55,56,57,58,59]. Overall, the outcomes after SBRT compared very favorably, without evidence of unanticipated increased late toxic effects. Menkarios et al. [46] reported 5% GU and 11% GI toxicity at approximately 2 years following IMRT and delivering 45 Gy in nine 5 Gy fractions, once weekly. In one study, grade ≥ 3 GI toxicities consisted of rectal bleeding with one patient experiencing hemorrhagic cystitis that required a blood transfusion and a radical cysto-prostatectomy for bladder necrosis. Similarly, Hannan et al. reported 6% GU and 7% GI grade ≥ 3 toxicity at 5 years. Late grade 3 GU toxicity consisted of cystitis requiring uretero-ileal diversion and was mainly observed in the 50 Gy dose escalation arm [47]. 

Chen et al. recently reviewed 249 low–intermediate- and high-PCa patients treated in different series with dose-escalated SBRT up to 45 Gy in five fractions. With a median follow-up of 14.9 months, late GU and GI grade ≥ 3 toxicity occurred in 6 and 1.5% of the patients, respectively [60]. 

It is possible that the relatively low incidence of severe late GI toxicity in our 50 Gy-treated patients was due to the placement of a rectal balloon spacer. Folkert et al. recently reported a series of 44 men treated with SBRT at 45 Gy in five fractions using a rectal balloon spacer and showed no incidence of grade 3 toxicity in any domain with an excellent biochemical control of 93% in intermediate-risk patients at a median 48-month follow-up [61]. 

To better put our results into context, we also compared different rates of self-reported patient perception of quality of life at baseline and at 3, 6, 9, and 12 months follow-up in different series, and acknowledging the differences in self-reported questionnaires used by the different trials, we observed that scores gradually decreased, reaching a plateau at 12 months and subsequently improving (Supplementary Table S2) [22,49,51,55,56].

The mechanisms of GU and GI toxicity are many and complex. They include patient-related characteristics such as baseline urine symptoms, prostate volume, the presence of the urethra in the high-dose-volume area, radiation delivery processes such as bladder and rectal filling during treatment, and dosimetric parameters. While our efforts to develop an innovative strategy succeeded quite satisfactorily, the precise dosimetric restrictions to prevent GU and GI toxicity are not well understood and are a subject of active investigation.

The use of a rectal spacer prior to 50 Gy DIN SBRT, as well as rectal and bladder/urethra limitations learned from our phase I/II trial experience, most likely contributed to lower rectal toxicity risks in our patients compared to previous publications that also attempted to deliver 50 Gy to the prostate.

## 5. Conclusions

Among patients with intermediate–high-risk PCa undergoing focal dose-escalated SBRT without ADT, DIN recurrences were occasional (12%). When present, these recurrences were typically located at the original site or adjacent to the initial tumor. Conversely, relapses beyond the DIN (9%) and in extra-prostatic (metastatic) sites were prevalent, underscoring the significance of systemic ADT in managing this patient population.

## Figures and Tables

**Table 1:** Tumor and Treatment Characteristics.

Characteristics	N (Total = 33)	%
**T stage**		
T1c	2	6.1
T2a	10	30.3
T2b	9	27.3
T2c	8	24.2
T3a	3	9.1
T3b	1	3
**Gleason Score**		
3 + 3	3	9.1
3 + 4	17	51.5
4 + 3	5	15.2
4 + 4	4	12.1
3 + 5	1	3
4 + 5	3	9.1
Low risk		
(GS < 6 and PSA < 10 and T1c/T2a)	1	3
Intermediate risk		
(GS = 7 or PSA > 10 or ≤cT2a)	14	42.4
High risk		
(GS ≥ 8 or ≥cT2c or PSA > 20)	18	54.6
**Hormonal therapy**		
Yes	1	3
No	32	97
**Radiotherapy delivery**		
CyberKnife	27	81.8
Tomotherapy	3	9.1
Synergy	3	9.1

Abbreviations: PSA, prostate-specific antigen; GS, Gleason score; T, tumor.

**Table 2:** Late genitourinary toxicity grade ≥ 2.

UPN	Grade 2	Grade 3
105	Nycturia	NR
106	Urinary urgency and pollakiuria	NR
107	Pollakiuria and urinary incontinence	NR
110	Pollakiuria, urinary urgency and incontinence	NR
111	Pollakiuria	NR
113	NR	Urinary obstruction
114	Urinary tract infection	NR

UPN, unique patient number; NR, not reported. Toxicity graded according to Common Terminology Criteria for Adverse Events, version 4. No grade ≥ 4 toxicity was reported. Time period: more than 90 days after first fraction of radiation therapy.

## Data Availability

Research data are stored in an institutional repository and will be shared upon request to the corresponding author.

## Supplementary Materials

The following supporting information can be downloaded at: https://www.mdpi.com/article/10.3390/cancers16112097/s1. Figure S1. Description of a patient’s case (T3a, GS 4 + 3, PSA baseline 12.6 μg/L) with mpMRI and ^68^Ga-PSMA-PET/CT at baseline and at recurrence. SBRT histograms. Evolution of PSA values. Supplementary Table S1: Dosimetry-related genitourinary and gastrointestinal toxicity. Supplementary Table S2: Dosimetric parameters and Quality of Life assessments.

## References

[1] Sung H., Ferlay J., Siegel R.L., Laversanne M., Soerjomataram I., Jemal A., Bray F. (2021). Global Cancer Statistics 2020: GLOBOCAN Estimates of Incidence and Mortality Worldwide for 36 Cancers in 185 Countries. CA A Cancer J. Clin..

[2] Widmark A., Klepp O., Solberg A., Damber J.-E., Angelsen A., Fransson P., Lund J.-Å., Tasdemir I., Hoyer M., Wiklund F. (2009). Endocrine Treatment, with or without Radiotherapy, in Locally Advanced Prostate Cancer (SPCG-7/SFUO-3): An Open Randomised Phase III Trial. Lancet.

[3] Bolla M., Van Tienhoven G., Warde P., Dubois J.B., Mirimanoff R.-O., Storme G., Bernier J., Kuten A., Sternberg C., Billiet I. (2010). External Irradiation with or without Long-Term Androgen Suppression for Prostate Cancer with High Metastatic Risk: 10-Year Results of an EORTC Randomised Study. Lancet Oncol..

[4] Warde P., Mason M., Ding K., Kirkbride P., Brundage M., Cowan R., Gospodarowicz M., Sanders K., Kostashuk E., Swanson G. (2011). Combined Androgen Deprivation Therapy and Radiation Therapy for Locally Advanced Prostate Cancer: A Randomised, Phase 3 Trial. Lancet.

[5] Peeters S.T.H., Heemsbergen W.D., Koper P.C.M., van Putten W.L.J., Slot A., Dielwart M.F.H., Bonfrer J.M.G., Incrocci L., Lebesque J.V. (2006). Dose-Response in Radiotherapy for Localized Prostate Cancer: Results of the Dutch Multicenter Randomized Phase III Trial Comparing 68 Gy of Radiotherapy with 78 Gy. J. Clin. Oncol..

[6] Dearnaley D.P., Sydes M.R., Graham J.D., Aird E.G., Bottomley D., Cowan R.A., Huddart R.A., Jose C.C., Matthews J.H., Millar J. (2007). Escalated-Dose versus Standard-Dose Conformal Radiotherapy in Prostate Cancer: First Results from the MRC RT01 Randomised Controlled Trial. Lancet Oncol..

[7] Morris W.J., Tyldesley S., Rodda S., Halperin R., Pai H., McKenzie M., Duncan G., Morton G., Hamm J., Murray N. (2017). Androgen Suppression Combined with Elective Nodal and Dose Escalated Radiation Therapy (the ASCENDE-RT Trial): An Analysis of Survival Endpoints for a Randomized Trial Comparing a Low-Dose-Rate Brachytherapy Boost to a Dose-Escalated External Beam Boost for High- and Intermediate-Risk Prostate Cancer. Int. J. Radiat. Oncol. Biol. Phys..

[8] Spratt D.E., Soni P.D., McLaughlin P.W., Merrick G.S., Stock R.G., Blasko J.C., Zelefsky M.J. (2017). American Brachytherapy Society Task Group Report: Combination of Brachytherapy and External Beam Radiation for High-Risk Prostate Cancer. Brachytherapy.

[9] Hoskin P.J., Rojas A.M., Ostler P.J., Bryant L., Lowe G.J. (2021). Randomised Trial of External-Beam Radiotherapy Alone or with High-Dose-Rate Brachytherapy for Prostate Cancer: Mature 12-Year Results. Radiother. Oncol..

[10] Dearnaley D., Syndikus I., Mossop H., Khoo V., Birtle A., Bloomfield D., Graham J., Kirkbride P., Logue J., Malik Z. (2016). Conventional versus Hypofractionated High-Dose Intensity-Modulated Radiotherapy for Prostate Cancer: 5-Year Outcomes of the Randomised, Non-Inferiority, Phase 3 CHHiP Trial. Lancet Oncol..

[11] Parr H., Porta N., Tree A.C., Dearnaley D., Hall E. (2023). A Personalized Clinical Dynamic Prediction Model to Characterize Prognosis for Patients with Localized Prostate Cancer: Analysis of the CHHiP Phase 3 Trial. Int. J. Radiat. Oncol. Biol. Phys..

[12] Catton C.N., Lukka H., Gu C.-S., Martin J.M., Supiot S., Chung P.W.M., Bauman G.S., Bahary J.-P., Ahmed S., Cheung P. (2017). Randomized Trial of a Hypofractionated Radiation Regimen for the Treatment of Localized Prostate Cancer. J. Clin. Oncol..

[13] Lee W.R., Dignam J.J., Amin M.B., Bruner D.W., Low D., Swanson G.P., Shah A.B., D’Souza D.P., Michalski J.M., Dayes I.S. (2016). Randomized Phase III Noninferiority Study Comparing Two Radiotherapy Fractionation Schedules in Patients with Low-Risk Prostate Cancer. J. Clin. Oncol..

[14] Boike T.P., Lotan Y., Cho L.C., Brindle J., DeRose P., Xie X.-J., Yan J., Foster R., Pistenmaa D., Perkins A. (2011). Phase I Dose-Escalation Study of Stereotactic Body Radiation Therapy for Low- and Intermediate-Risk Prostate Cancer. J. Clin. Oncol..

[15] Kim D.W.N., Cho L.C., Straka C., Christie A., Lotan Y., Pistenmaa D., Kavanagh B.D., Nanda A., Kueplian P., Brindle J. (2014). Predictors of Rectal Tolerance Observed in a Dose-Escalated Phase 1-2 Trial of Stereotactic Body Radiation Therapy for Prostate Cancer. Int. J. Radiat. Oncol. Biol. Phys..

[16] Arrayeh E., Westphalen A.C., Kurhanewicz J., Roach M., Jung A.J., Carroll P.R., Coakley F.V. (2012). Does Local Recurrence of Prostate Cancer After Radiation Therapy Occur at the Site of Primary Tumor? Results of a Longitudinal MRI and MRSI Study. Int. J. Radiat. Oncol. Biol. Phys..

[17] Cellini N., Morganti A.G., Mattiucci G.C., Valentini V., Leone M., Luzi S., Manfredi R., Dinapoli N., Digesu’ C., Smaniotto D. (2002). Analysis of Intraprostatic Failures in Patients Treated with Hormonal Therapy and Radiotherapy: Implications for Conformal Therapy Planning. Int. J. Radiat. Oncol. Biol. Phys..

[18] Herrera F.G., Valerio M., Berthold D., Tawadros T., Meuwly J.-Y., Vallet V., Baumgartner P., Thierry A.-C., De Bari B., Jichlinski P. (2019). 50-Gy Stereotactic Body Radiation Therapy to the Dominant Intraprostatic Nodule: Results From a Phase 1a/b Trial. Int. J. Radiat. Oncol. Biol. Phys..

[19] Puech P., Sufana Iancu A., Renard B., Villers A., Lemaitre L. (2012). Detecting Prostate Cancer with MRI—Why and How. Diagn. Interv. Imaging.

[20] D’Amico A.V., Whittington R., Malkowicz S.B., Schultz D., Blank K., Broderick G.A., Tomaszewski J.E., Renshaw A.A., Kaplan I., Beard C.J. (1998). Biochemical Outcome after Radical Prostatectomy, External Beam Radiation Therapy, or Interstitial Radiation Therapy for Clinically Localized Prostate Cancer. JAMA.

[21] Collins S., Lei S., Piel N., Oermann E., Chen V., Ju A., Dahal K., Hanscom H., Kim J., Yu X. (2011). Six-Dimensional Correction of Intra-Fractional Prostate Motion with CyberKnife Stereotactic Body Radiation Therapy. Front. Oncol..

[22] Cloitre M., Valerio M., Mampuya A., Rakauskas A., Berthold D., Tawadros T., Meuwly J.-Y., Heym L., Duclos F., Vallet V. (2023). Toxicity, Quality of Life, and PSA Control after 50 Gy Stereotactic Body Radiation Therapy to the Dominant Intraprostatic Nodule with the Use of a Rectal Spacer: Results of a Phase I/II Study. BJR.

[23] Le Tourneau C., Lee J.J., Siu L.L. (2009). Dose Escalation Methods in Phase I Cancer Clinical Trials. J. Natl. Cancer Inst..

[24] Simon R. (1989). Optimal Two-Stage Designs for Phase II Clinical Trials. Control. Clin. Trials.

[25] Fayers P.M. (2001). Interpreting Quality of Life Data: Population-Based Reference Data for the EORTC QLQ-C30. Eur. J. Cancer.

[26] Osoba D., Rodrigues G., Myles J., Zee B., Pater J. (1998). Interpreting the Significance of Changes in Health-Related Quality-of-Life Scores. J. Clin. Oncol..

[27] Roach M., Hanks G., Thames H., Schellhammer P., Shipley W.U., Sokol G.H., Sandler H. (2006). Defining Biochemical Failure Following Radiotherapy with or without Hormonal Therapy in Men with Clinically Localized Prostate Cancer: Recommendations of the RTOG-ASTRO Phoenix Consensus Conference. Int. J. Radiat. Oncol. Biol. Phys..

[28] Abghari-Gerst M., Armstrong W.R., Nguyen K., Calais J., Czernin J., Lin D., Jariwala N., Rodnick M., Hope T.A., Hearn J. (2022). A Comprehensive Assessment of ^68^Ga-PSMA-11 PET in Biochemically Recurrent Prostate Cancer: Results from a Prospective Multi-Center Study in 2005 Patients. J. Nucl. Med..

[29] Barrier A., Ouzzane A., Villers A. (2017). Rôle de l’IRM Prostatique Dans Le Cancer de La Prostate En 2016: Mise Au Point et Perspectives d’avenir. Afr. J. Urol..

[30] Kishan A.U., Dang A., Katz A.J., Mantz C.A., Collins S.P., Aghdam N., Chu F.-I., Kaplan I.D., Appelbaum L., Fuller D.B. (2019). Long-Term Outcomes of Stereotactic Body Radiotherapy for Low-Risk and Intermediate-Risk Prostate Cancer. JAMA Netw. Open.

[31] Groen V.H., Haustermans K., Pos F.J., Draulans C., Isebaert S., Monninkhof E.M., Smeenk R.J., Kunze-Busch M., de Boer J.C.J., van der Voort van Zijp J. (2022). Patterns of Failure Following External Beam Radiotherapy with or without an Additional Focal Boost in the Randomized Controlled FLAME Trial for Localized Prostate Cancer. Eur. Urol..

[32] Kerkmeijer L.G.W., Groen V.H., Pos F.J., Haustermans K., Monninkhof E.M., Smeenk R.J., Kunze-Busch M., de Boer J.C.J., van der Voort van Zijp J., van Vulpen M. (2021). Focal Boost to the Intraprostatic Tumor in External Beam Radiotherapy for Patients with Localized Prostate Cancer: Results from the FLAME Randomized Phase III Trial. J. Clin. Oncol..

[33] Jackson W.C., Silva J., Hartman H.E., Dess R.T., Kishan A.U., Beeler W.H., Gharzai L.A., Jaworski E.M., Mehra R., Hearn J.W.D. (2019). Stereotactic Body Radiation Therapy for Localized Prostate Cancer: A Systematic Review and Meta-Analysis of Over 6,000 Patients Treated On Prospective Studies. Int. J. Radiat. Oncol. Biol. Phys..

[34] Widmark A., Gunnlaugsson A., Beckman L., Thellenberg-Karlsson C., Hoyer M., Lagerlund M., Kindblom J., Ginman C., Johansson B., Björnlinger K. (2019). Ultra-Hypofractionated versus Conventionally Fractionated Radiotherapy for Prostate Cancer: 5-Year Outcomes of the HYPO-RT-PC Randomised, Non-Inferiority, Phase 3 Trial. Lancet.

[35] Meier R.M., Bloch D.A., Cotrutz C., Beckman A.C., Henning G.T., Woodhouse S.A., Williamson S.K., Mohideen N., Dombrowski J.J., Hong R.L. (2018). Multicenter Trial of Stereotactic Body Radiation Therapy for Low- and Intermediate-Risk Prostate Cancer: Survival and Toxicity Endpoints. Int. J. Radiat. Oncol. Biol. Phys..

[36] Macias V.A., Blanco M.L., Barrera I., Garcia R. (2014). A Phase II Study of Stereotactic Body Radiation Therapy for Low-Intermediate-High-Risk Prostate Cancer Using Helical Tomotherapy: Dose-Volumetric Parameters Predicting Early Toxicity. Front. Oncol..

[37] Bolzicco G., Favretto M.S., Satariano N., Scremin E., Tambone C., Tasca A. (2013). A Single-Center Study of 100 Consecutive Patients with Localized Prostate Cancer Treated with Stereotactic Body Radiotherapy. BMC Urol..

[38] Tree A.C., Ostler P., Hoskin P., Dankulchai P., Nariyangadu P., Hughes R.J., Wells E., Taylor H., Khoo V.S., van As N.J. (2014). Prostate Stereotactic Body Radiotherapy—First UK Experience. Clin. Oncol..

[39] Fan C.-Y., Chao H.-L., Huang W.-Y., Lin C.-S., Chen C.-M., Lo C.-H. (2015). Stereotactic Ablative Radiotherapy with CyberKnife in the Treatment of Locally Advanced Prostate Cancer: Preliminary Results. Tumori J..

[40] Kotecha R., Djemil T., Tendulkar R.D., Reddy C.A., Thousand R.A., Vassil A., Stovsky M., Berglund R.K., Klein E.A., Stephans K.L. (2016). Dose-Escalated Stereotactic Body Radiation Therapy for Patients with Intermediate- and High-Risk Prostate Cancer: Initial Dosimetry Analysis and Patient Outcomes. Int. J. Radiat. Oncol. Biol. Phys..

[41] King C.R., Freeman D., Kaplan I., Fuller D., Bolzicco G., Collins S., Meier R., Wang J., Kupelian P., Steinberg M. (2013). Stereotactic Body Radiotherapy for Localized Prostate Cancer: Pooled Analysis from a Multi-Institutional Consortium of Prospective Phase II Trials. Radiother. Oncol..

[42] Katz A., Kang J. (2014). Stereotactic Body Radiotherapy with or without External Beam Radiation as Treatment for Organ Confined High-Risk Prostate Carcinoma: A Six Year Study. Radiat. Oncol..

[43] Spratt D.E., Pei X., Yamada J., Kollmeier M.A., Cox B., Zelefsky M.J. (2013). Long-Term Survival and Toxicity in Patients Treated with High-Dose Intensity Modulated Radiation Therapy for Localized Prostate Cancer. Int. J. Radiat. Oncol. Biol. Phys..

[44] Ricco A., Hanlon A., Lanciano R. (2017). Propensity Score Matched Comparison of Intensity Modulated Radiation Therapy vs Stereotactic Body Radiation Therapy for Localized Prostate Cancer: A Survival Analysis from the National Cancer Database. Front. Oncol..

[45] van Dams R., Jiang N.Y., Fuller D.B., Loblaw A., Jiang T., Katz A.J., Collins S.P., Aghdam N., Suy S., Stephans K.L. (2021). Stereotactic Body Radiotherapy for High-Risk Localized Carcinoma of the Prostate (SHARP) Consortium: Analysis of 344 Prospectively Treated Patients. Int. J. Radiat. Oncol. Biol. Phys..

[46] Menkarios C., Vigneault É., Brochet N., Nguyen D.H., Bahary J.-P., Jolicoeur M., Beauchemin M.-C., Villeneuve H., Van Nguyen T., Fortin B. (2011). Toxicity Report of Once Weekly Radiation Therapy for Low-Risk Prostate Adenocarcinoma: Preliminary Results of a Phase I/II Trial. Radiat. Oncol..

[47] Hannan R., Tumati V., Xie X.-J., Cho L.C., Kavanagh B.D., Brindle J., Raben D., Nanda A., Cooley S., Kim D.W.N. (2016). Stereotactic Body Radiation Therapy for Low and Intermediate Risk Prostate Cancer—Results from a Multi-Institutional Clinical Trial. Eur. J. Cancer.

[48] Katz A.J., Santoro M., Diblasio F., Ashley R. (2013). Stereotactic Body Radiotherapy for Localized Prostate Cancer: Disease Control and Quality of Life at 6 Years. Radiat. Oncol..

[49] Musunuru H.B., D’Alimonte L., Davidson M., Ho L., Cheung P., Vesprini D., Liu S., Chu W., Chung H., Ravi A. (2018). Phase 1-2 Study of Stereotactic Ablative Radiotherapy Including Regional Lymph Node Irradiation in Patients With High-Risk Prostate Cancer (SATURN): Early Toxicity and Quality of Life. Int. J. Radiat. Oncol. Biol. Phys..

[50] Shikama N., Kumazaki Y., Miyazawa K., Nihei K., Hashimoto S., Tsukamoto N. (2016). Rectal Toxicity After Extremely Hypofractionated Radiotherapy Using a Non-Isocentric Robotic Radiosurgery System for Early Stage Prostate Cancer. World J. Oncol..

[51] Chen L.N., Suy S., Uhm S., Oermann E.K., Ju A.W., Chen V., Hanscom H.N., Laing S., Kim J.S., Lei S. (2013). Stereotactic Body Radiation Therapy (SBRT) for Clinically Localized Prostate Cancer: The Georgetown University Experience. Radiat. Oncol..

[52] Murthy V., Gupta M., Mulye G., Maulik S., Munshi M., Krishnatry R., Phurailatpam R., Mhatre R., Prakash G., Bakshi G. (2018). Early Results of Extreme Hypofractionation Using Stereotactic Body Radiation Therapy for High-Risk, Very High-Risk and Node-Positive Prostate Cancer. Clin. Oncol..

[53] Alayed Y., Cheung P., Vesprini D., Liu S., Chu W., Chung H., Musunuru H.B., Davidson M., Ravi A., Ho L. (2019). SABR in High-Risk Prostate Cancer: Outcomes From 2 Prospective Clinical Trials With and Without Elective Nodal Irradiation. Int. J. Radiat. Oncol. Biol. Phys..

[54] Aluwini S., van Rooij P., Hoogeman M., Bangma C., Kirkels W.J., Incrocci L., Kolkman-Deurloo I.-K. (2010). CyberKnife Stereotactic Radiotherapy as Monotherapy for Low- to Intermediate-Stage Prostate Cancer: Early Experience, Feasibility, and Tolerance. J. Endourol..

[55] Elias E., Helou J., Zhang L., Cheung P., Deabreu A., D’Alimonte L., Sethukavalan P., Mamedov A., Cardoso M., Loblaw A. (2014). Dosimetric and Patient Correlates of Quality of Life after Prostate Stereotactic Ablative Radiotherapy. Radiother. Oncol..

[56] Vargas C.E., Hartsell W.F., Dunn M., Keole S.R., Doh L., Chang J., Larson G.L. (2016). Image-Guided Hypofractionated Proton Beam Therapy for Low-Risk Prostate Cancer: Analysis of Quality of Life and Toxicity, PCG GU 002. Rep. Pract. Oncol. Radiother..

[57] Madsen B.L., Hsi R.A., Pham H.T., Fowler J.F., Esagui L., Corman J. (2007). Stereotactic Hypofractionated Accurate Radiotherapy of the Prostate (SHARP), 33.5 Gy in Five Fractions for Localized Disease: First Clinical Trial Results. Int. J. Radiat. Oncol. Biol. Phys..

[58] Alongi F., Cozzi L., Arcangeli S., Iftode C., Comito T., Villa E., Lobefalo F., Navarria P., Reggiori G., Mancosu P. (2013). Linac Based SBRT for Prostate Cancer in 5 Fractions with VMAT and Flattening Filter Free Beams: Preliminary Report of a Phase II Study. Radiat. Oncol..

[59] Głowacki G., Majewski W., Wojcieszek P., Grabinska K., Wozniak G., Miszczyk L. (2017). Ultrahypofractionated CyberKnifeTM Based Stereotactic Radiotherapy versus Conventional Radiotherapy in Patients with Prostate Cancer–Acute Toxicity Evaluation in Two Phase II Prospective Studies. NEO.

[60] Chen L., Gannavarapu B.S., Desai N.B., Folkert M.R., Dohopolski M., Gao A., Ahn C., Cadeddu J., Bagrodia A., Woldu S. (2022). Dose-Intensified Stereotactic Ablative Radiation for Localized Prostate Cancer. Front. Oncol..

[61] Folkert M.R., Zelefsky M.J., Hannan R., Desai N.B., Lotan Y., Laine A.M., Kim D.W.N., Neufeld S.H., Hornberger B., Kollmeier M.A. (2021). A Multi-Institutional Phase 2 Trial of High-Dose SAbR for Prostate Cancer Using Rectal Spacer. Int. J. Radiat. Oncol. Biol. Phys..

