# Correction: Cloître et al. Spatial Distribution of Recurrence and Long-Term Toxicity Following Dose Escalation to the Dominant Intra-Prostatic Nodule for Intermediate–High-Risk Prostate Cancer: Insights from a Phase I/II Study. *Cancers* 2024, *16*, 2097

**DOI:** 10.3390/cancers17152496

**Published:** 2025-07-29

**Authors:** Minna Cloître, Sofian Benkhaled, Sarah Boughdad, Niklaus Schaefer, John O. Prior, Michele Zeverino, Dominik Berthold, Thomas Tawadros, Jean-Yves Meuwly, Paul Martel, Chantal Rohner, Leonie Heym, Frederic Duclos, Véronique Vallet, Massimo Valerio, Jean Bourhis, Fernanda G. Herrera

**Affiliations:** 1 Department of Oncology, Radiation Oncology Service, Centre Hospitalier Universitaire Vaudois, 1005 Lausanne, Switzerland; minna.cloitre@chuv.ch (M.C.); sofian.benkhaled@chuv.ch (S.B.); michele.zeverino@chuv.ch (M.Z.); leonie.heym@chuv.ch (L.H.); frederic.duclos@chuv.ch (F.D.); veronique.vallet@chuv.ch (V.V.); jean.bourhis@chuv.ch (J.B.); 2 Department of Medical Imaging, Nuclear Medicine Service, Centre Hospitalier Universitaire Vaudois, 1005 Lausanne, Switzerland; sarah.boughdad@chuv.ch (S.B.); niklaus.schaefer@chuv.ch (N.S.);; 3 Department of Oncology, Medical Oncology Service, Centre Hospitalier Universitaire Vaudois, 1005 Lausanne, Switzerland; dominik.berthold@chuv.ch; 4 Department of Surgery, Urology Service, Centre Hospitalier Universitaire Vaudois, 1005 Lausanne, Switzerland; thomas.tawadros@hopitalrivierachablais.ch (T.T.); paul.martel@chuv.ch (P.M.); massimo.valerio@hcuge.ch (M.V.); 5 Department of Medical Imaging, Radiology Service, Centre Hospitalier Universitaire Vaudois, 1005 Lausanne, Switzerland; jean-yves.meuwly@chuv.ch (J.-Y.M.); chantal.rohner@chuv.ch (C.R.); 6 Ludwig Cancer Research Center Lausanne, 1005 Lausanne, Switzerland

## Error in Figure

In the original publication [1], there was a mistake in Figure 2: Locoregional recurrences in individual patients as published. The conversion of the figure into various formats altered the presentation of the figure and rendered it less clear. The corrected Figure 2 appears below.

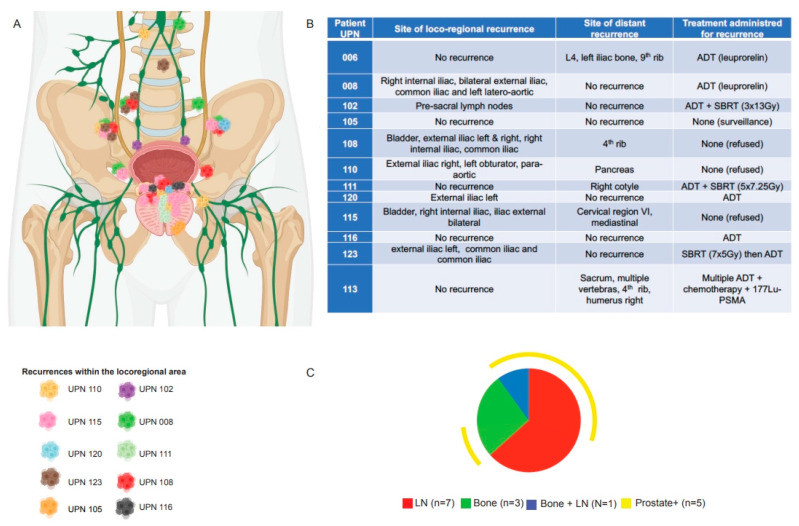



## Additional Affiliation

In the original publication [1], there was an error regarding the affiliation for Chantal Rohner. Instead of affiliation 4, there should be 5. Besides this, the corresponding author’s name should be corrected to Fernanda G. Herrera.

## Missing Citations

In the original publication [1], Herrera et al. 2019 was not cited. The citation has now been inserted in 1. Introduction, Paragraph 6 and should read as follows:

“We previously reported a prospective phase I/II study, in which we used SBRT to irradiate the whole prostate gland with tumoricidal doses of 36.25 Gy in five fractions while simultaneously increasing the RT dose to the DIN up to 50 Gy [18].”

In the original publication [1], Cloitre et al. 2023 was not cited. The citation has now been inserted in 2. Materials and Methods, 2.1. Radiotherapy Planning and Delivery, Paragraph 4, and should read as follows:

“Dose–volume histogram goals and RT planning details were previously published [22]. Briefly, dose–volume histogram goals for the rectum included a maximum dose of 0.1 cm^3^ < 41 Gy and V25 < 20 (volume receiving 25 Gy < 20 cm^3^). Bladder dose–volume histogram limits required <0.1 cm^3^ to receive <45 Gy, and the bladder median dose was capped at 20 Gy. Urethra dose was restricted to <1 cm^3^ of urethra receiving >39 Gy, with <0.1 cm^3^ not to exceed 41 Gy.”

In the original publication [1], Cloitre et al. 2023 was not cited. The citation has now been inserted in 2. Materials and Methods, 2.3. Phase I/II Design, Paragraph 2, and should read as follows:

“Primary endpoint results (safety) and secondary endpoints including biochemical control and quality of life were recently published elsewhere [22].”

In the original publication [1], not all citations of Supplementary Table S1 were cited, although cited elsewhere in the Discussion. The citation has now been inserted in 4. Discussion, Paragraph 6, and should read as follows:

“Due to the rising concerns associated with clinical observations of significant late toxicity in other dose escalation trials [14], we present data with a long-term follow-up focused on treatment safety. Although late grade 3 toxicity remained low (3% GU and 6% GI) in this study, we demonstrated a significant association between the volume of irradiation and the risk of late toxicity in patients treated with dose-escalated SBRT. To place the results of the present series in the appropriate context, we extracted the rates of late severe (i.e., grade 3 or higher) toxic events after treatment reported in other series a with long-term follow-up (Supplementary Table S1) [22,38,42,46–59]. Overall, the outcomes after SBRT compared very favorably, without evidence of unanticipated increased late toxic effects. Menkarios et al. [46] reported 5% GU and 11% GI toxicity at approximately 2 years following IMRT and delivering 45 Gy in nine 5 Gy fractions, once weekly. In one study, grade ≥ 3 GI toxicities consisted of rectal bleeding with one patient experiencing hemorrhagic cystitis that required a blood transfusion and a radical cysto-prostatectomy for bladder necrosis. Similarly, Hannan et al. reported 6% GU and 7% GI grade ≥ 3 toxicity at 5 years. Late grade 3 GU toxicity consisted of cystitis requiring uretero-ileal diversion and was mainly observed in the 50 Gy dose escalation arm [47].”

In the original publication [1], not all citations of Supplementary Table S2 were cited, although cited elsewhere in the Discussion. The citation has now been inserted in 4. Discussion, Paragraph 9, and should read as follows:

“To better put our results into context, we also compared different rates of self-reported patient perception of quality of life at baseline and at 3, 6, 9, and 12 months follow-up in different series, and acknowledging the differences in self-reported questionnaires used by the different trials, we observed that scores gradually decreased, reaching a plateau at 12 months and subsequently improving (Supplementary Table S2) [22,49,51,55,56].”

## Text Correction

There was an error in the original publication [1]. The initials of the involved physicist, originally not included due to anonymity, were not corrected after approval.

A correction has been made to 2. Materials and Methods, 2.1. Radiotherapy Planning and Delivery, Paragraph 2:

“Following the insertion of rectal spacer/fiducial markers, planning T2-weighted MRI image sets were rigidly fused to planning CT images (fiducials-based registration), omitting the use of a catheter for urethral visualization. This method was employed to ensure contouring accuracy and appropriate visualization of the DIN and organs at risk (OARs). To minimize prostate motion during planning scans and treatment, several precautions were implemented. Patients were advised to adhere to a low-fiber diet starting 5 days before planning scans until treatment completion to reduce intestinal gas. A moderate laxative was administered 48 h prior to planned MRI and CT scans. Enemas were performed if necessary 1 h before planning scans and before each treatment session to minimize rectal volume. Patients were encouraged to drink 200 mL of water 1 h before scans, following complete voiding. Anatomical contours of the prostate, DIN, seminal vesicles, and OARs were delineated by lead investigators (F.H. and J.B.), then reviewed by a panel of board-certified radiation oncologists. Identification of the DIN and urethra as the region of interest on MRI was conducted by a radiologist (J.-Y.M.). The planned target volume was generated by uniformly expanding the prostate by 3 mm (PTVp). The DIN was outlined as the gross tumor volume (GTV) and extended by 3 mm to form a PTVDIN, without employing a clinical target volume around the GTV. The prescribed dose to the PTVp was 36.25 Gy administered in 5 fractions (7.25 Gy per fraction) at the 80% isodose line.”

There was an error in the original publication [1]. The initials of the involved physicist, originally not included due to anonymity, were not corrected after approval.

A correction has been made to 2. Materials and Methods, 2.4. Determination of Recurrences, Paragraph 1:

“Patients were monitored with PSA measurements and physical examinations every 3 months for the first 2 years, and every 6 months thereafter. Biochemical recurrence was determined using the nadir +2 μg/mL failure definition [27]. Upon confirmation of biochemical recurrence, patients were staged using 68Ga-PSMA-PET/CT and prostate mpMRI, which were evaluated by dedicated and experienced nuclear medicine physicians (J.P., S.B., N.S.) and radiologists (J.-Y.M.) in order to determine the exact site of failure. Any focal 68Ga-PSMA-11 uptake above location-specific background levels was considered PSMA-positive. This is the definition according to the largest prospective analysis of PSMA-11 in over 2000 patients [28]. We determine the distance between the DIN and the urethra/rectum and its associated grade ≥ 2 toxicity. Results were considered significative with a *p*-value ≤ 0.05 using Welch’s correction. Analyses were conducted in Prism 7 (GraphPad Software, Inc., La Jolla, CA, USA).”

## Newly Added Citations and References

18. Herrera, F.G.; Valerio, M.; Berthold, D.; Tawadros, T.; Meuwly, J.-Y.; Vallet, V.; Baumgartner, P.; Thierry, A.-C.; De Bari, B.; Jichlinski, P.; et al. 50-Gy Stereotactic Body Radiation Therapy to the Dominant Intraprostatic Nodule: Results From a Phase 1a/b Trial. *Int. J. Radiat. Oncol. Biol. Phys.* **2019**, *103*, 320–334. https://doi.org/10.1016/j.ijrobp.2018.09.023.
22. Cloitre, M.; Valerio, M.; Mampuya, A.; Rakauskas, A.; Berthold, D.; Tawadros, T.; Meuwly, J.-Y.; Heym, L.; Duclos, F.; Vallet, V.; et al. Toxicity, Quality of Life, and PSA Control after 50 Gy Stereotactic Body Radiation Therapy to the Dominant Intraprostatic Nodule with the Use of a Rectal Spacer: Results of a Phase I/II Study. *BJR* **2023**, *96*, 20220803. https://doi.org/10.1259/bjr.20220803.
48. Katz, A.J.; Santoro, M.; Diblasio, F.; Ashley, R. Stereotactic Body Radiotherapy for Localized Prostate Cancer: Disease Control and Quality of Life at 6 Years. *Radiat. Oncol.* **2013**, *8*, 118. https://doi.org/10.1186/1748-717X-8-118.
49. Musunuru, H.B.; D’Alimonte, L.; Davidson, M.; Ho, L.; Cheung, P.; Vesprini, D.; Liu, S.; Chu, W.; Chung, H.; Ravi, A.; et al. Phase 1-2 Study of Stereotactic Ablative Radiotherapy Including Regional Lymph Node Irradiation in Patients With High-Risk Prostate Cancer (SATURN): Early Toxicity and Quality of Life. *Int. J. Radiat. Oncol. Biol. Phys.* **2018**, *102*, 1438–1447. https://doi.org/10.1016/j.ijrobp.2018.07.2005.
50. Shikama, N.; Kumazaki, Y.; Miyazawa, K.; Nihei, K.; Hashimoto, S.; Tsukamoto, N. Rectal Toxicity After Extremely Hypofractionated Radiotherapy Using a Non-Isocentric Robotic Radiosurgery System for Early Stage Prostate Cancer. *World J. Oncol.* **2016**, *7*, 98–103. https://doi.org/10.14740/wjon986w.
51. Chen, L.N.; Suy, S.; Uhm, S.; Oermann, E.K.; Ju, A.W.; Chen, V.; Hanscom, H.N.; Laing, S.; Kim, J.S.; Lei, S.; et al. Stereotactic Body Radiation Therapy (SBRT) for Clinically Localized Prostate Cancer: The Georgetown University Experience. *Radiat. Oncol.* **2013**, *8*, 58. https://doi.org/10.1186/1748-717X-8-58.
52. Murthy, V.; Gupta, M.; Mulye, G.; Maulik, S.; Munshi, M.; Krishnatry, R.; Phurailatpam, R.; Mhatre, R.; Prakash, G.; Bakshi, G. Early Results of Extreme Hypofractionation Using Stereotactic Body Radiation Therapy for High-Risk, Very High-Risk and Node-Positive Prostate Cancer. *Clin. Oncol.* **2018**, *30*, 442–447. https://doi.org/10.1016/j.clon.2018.03.004.
53. Alayed, Y.; Cheung, P.; Vesprini, D.; Liu, S.; Chu, W.; Chung, H.; Musunuru, H.B.; Davidson, M.; Ravi, A.; Ho, L.; et al. SABR in High-Risk Prostate Cancer: Outcomes From 2 Prospective Clinical Trials With and Without Elective Nodal Irradiation. *Int. J. Radiat. Oncol. Biol. Phys.* **2019**, *104*, 36–41. https://doi.org/10.1016/j.ijrobp.2018.11.011.
54. Aluwini, S.; van Rooij, P.; Hoogeman, M.; Bangma, C.; Kirkels, W.J.; Incrocci, L.; Kolkman-Deurloo, I.-K. CyberKnife Stereotactic Radiotherapy as Monotherapy for Low- to Intermediate-Stage Prostate Cancer: Early Experience, Feasibility, and Tolerance. *J. Endourol.* **2010**, *24*, 865–869. https://doi.org/10.1089/end.2009.0438.
55. Elias, E.; Helou, J.; Zhang, L.; Cheung, P.; Deabreu, A.; D’Alimonte, L.; Sethukavalan, P.; Mamedov, A.; Cardoso, M.; Loblaw, A. Dosimetric and Patient Correlates of Quality of Life after Prostate Stereotactic Ablative Radiotherapy. *Radiother. Oncol.* **2014**, *112*, 83–88. https://doi.org/10.1016/j.radonc.2014.06.009.
56. Vargas, C.E.; Hartsell, W.F.; Dunn, M.; Keole, S.R.; Doh, L.; Chang, J.; Larson, G.L. Image-Guided Hypofractionated Proton Beam Therapy for Low-Risk Prostate Cancer: Analysis of Quality of Life and Toxicity, PCG GU 002. *Rep. Pract. Oncol. Radiother.* **2016**, *21*, 207–212. https://doi.org/10.1016/j.rpor.2016.01.002.
57. Madsen, B.L.; Hsi, R.A.; Pham, H.T.; Fowler, J.F.; Esagui, L.; Corman, J. Stereotactic Hypofractionated Accurate Radiotherapy of the Prostate (SHARP), 33.5 Gy in Five Fractions for Localized Disease: First Clinical Trial Results. *Int. J. Radiat. Oncol. Biol. Phys.* **2007**, *67*, 1099–1105. https://doi.org/10.1016/j.ijrobp.2006.10.050.
58. Alongi, F.; Cozzi, L.; Arcangeli, S.; Iftode, C.; Comito, T.; Villa, E.; Lobefalo, F.; Navarria, P.; Reggiori, G.; Mancosu, P.; et al. Linac Based SBRT for Prostate Cancer in 5 Fractions with VMAT and Flattening Filter Free Beams: Preliminary Report of a Phase II Study. *Radiat. Oncol.* **2013**, *8*, 171. https://doi.org/10.1186/1748-717X-8-171.
59. Głowacki, G.; Majewski, W.; Wojcieszek, P.; Grabinska, K.; Wozniak, G.; Miszczyk, L. Ultrahypofractionated CyberKnifeTM Based Stereotactic Radiotherapy versus Conventional Radiotherapy in Patients with Prostate Cancer–Acute Toxicity Evaluation in Two Phase II Prospective Studies. *NEO* **2017**, *64*, 599–604. https://doi.org/10.4149/neo_2017_421.

With this correction, the order of some references has been adjusted accordingly, Supplementary Material also updated. The authors apologize for any inconvenience caused and state that the scientific conclusions are unaffected. This correction was approved by the Academic Editor. The original publication has also been updated.
