# Supplementary material for: Spatial Distribution of Recurrence and Long-Term Toxicity Following Dose Escalation to the Dominant Intra-Prostatic Nodule for Intermediate–High-Risk Prostate Cancer: Insights from a Phase I/II Study
Source: Cancers (Basel). 2024 May 31;16(11):2097. doi: 10.3390/cancers16112097 (PMC11171188; doi:10.3390/cancers16112097)
Supplement: Supplementary file 1 [file cancers-16-02097-s001.zip › Suppl_Table_S1.pdf]

Supplementary Table S1: Dosimetry-related genito-urinary and gastro-intestinal toxicity

Author	Treatment technique	Bladder dose per protocol	Rectum dose per protocol	PTV dose per protocol	Median follow-up (range)	Toxicity GU (acute)		Toxicity GU (late)		Toxicity GI (acute)		Toxicitiy GI (late)	
						Grade I-II	Grade ≥ III	Grade I-II	Grade ≥ III	Grade I-II	Grade ≥ III	Grade I-II	Grade ≥ III
Cloitre et al. [22]	SBRT + boost to DIN	0.1 cm^3 < 45 Gy<br>Median dose < 20 Gy	0.1cm^3 < 41Gy<br>V25Gy < 20cm^3	Dmean $_{DIN}$ > 54Gy<br>D95%$_{DIN}$ >47.5Gy<br>Dmax $_{DIN}$ = 62.5 Gy	79 months<br>(66-127)	72.5%<br>(57.5% G I)	0%	78.7%<br>(66.6% G I)	3%	30.3%<br>(24.2% G I)	0%	18.1%<br>(15.1% G I)	6%
Musunru et al. (SATURN) [49]	SABR + boost to pelvis	V32Gy <10%<br>V35Gy <5%	V32Gy <10%<br>V35Gy <5%	V23.75Gy >99%<br>V33.25Gy >99%	26 months<br>(18-31)	46.7%<br>(Grade II)	0%	52%<br>(Grade II)	0%	3.3%<br>(Grade II)	0%	32%<br>(Grade II)	0%
Shikama et al. [50]	SBRT	D1cm^3 <38Gy<br>D10% <31.5Gy<br>D50% <17.5Gy	D1cm^3 <36.75Gy<br>D3cm^3 <33.25Gy<br>D10% <31.5Gy<br>D20% <28Gy	D1cm^3 < 42Gy<br>D98% >33.25Gy<br>D99% >35Gy	30 months<br>(18-36)	NA	NA	NA	0%	55%	0%	15%	5%
Chen et al. (Georgetown) [51]	SBRT	V37Gy <5 cm^3	V36Gy <1 cm^3	V36.25Gy ≥95%	28 months<br>(17-42)	48%	0%	45%	0%	21%	0%	27%	0%
Murthy et al. [52]	SBRT ± boost to pelvis	V17.5Gy <20%<br>V35Gy <3%	V17.5Gy <40%<br>V28Gy <15%<br>V31.5Gy <8%<br>V35Gy <3%	D97% >36.4Gy	18 months	53%	0%	20.5%	4%	15%	0%	14%	0%
Katz et al. (2014) [42]	SBRT	V27.2 Gy ≤4cc<br>Mean D50% <15 Gy	V27.2 Gy ≤3cc<br>Mean D50% <15.8 Gy	D95% >31.5 Gy	60 months<br>(8-84)	NA	NA	7.8%<br>(Grade II)	3.9%	NA	NA	0%	0%
Alayed et al. [53]	pHART8:<br>SBRT + boost to SVs	V31.8Gy <15%	V28Gy <20%<br>V31.8Gy <15%	V28.5Gy >99% (PTV1)<br>V38Gy >99% (PTV2)	67 months	NA	NA	93.34%	0%	NA	NA	90%	3.33%
	SATURN:<br>SBRT + boost to SV & pelvis	V28Gy <15%	V28Gy <15%<br>V35Gy <5%	23.75Gy to PTV1<br>33.25Gy to PTV2	48 months	NA	NA	90%	0%	NA	NA	60%	0%
Glowacki et al. [59]	SBRT	V18Gy <55%<br>V29Gy <25%<br>V32.6Gy <15%<br>V36.25Gy <10%	V18Gy <50%<br>V29Gy <20%<br>V32.6Gy <10%<br>V36.25Gy <5%	V43.5Gy >99%	NA	56%	3%	NA	NA	29%	0%	NA	NA
Aluwini et al. [54]	SBRT	D1cm^3 ≤38Gy<br>Dmax ≤41.8Gy	D1cm^3 ≤32.3Gy<br>Dmax ≤28.5Gy<br>Dmax rectal wall ≤38Gy	V38Gy >99%	5 months<br>(2-13)	70%	0%	37%	0%	35%	0%	0%	0%
Elias et al. [55]	IMRT	V32Gy ≤40%<br>D5cm^3 ≤34Gy	V28Gy ≤40%<br>V32Gy ≤33%<br>D5cm^3 ≤33Gy	V33.25 ≥99%<br>Dmax ≤105%	51 months<br>(45-56)	20.4%<br>(Grade II)	0%	6%<br>(Grade II)	1.2%	9.5%<br>(Grade II)	0%	7.1%<br>(Grade II)	0%
Alongi et al. [58]	SBRT	D1% ≤34.1Gy	V32Gy ≤3.1%<br>D1% ≤33Gy	V33.25Gy ≥98.3%<br>D99% ≥33.1 Gy	11 months<br>(5-16)	60%	0%	16%	0%	25%	0%	0%	0%
Menkarios et al. [46]	3D-CRT	V49Gy ≤15%<br>V45Gy ≤30%<br>V40Gy ≤50%	V46Gy ≤15%<br>V43Gy ≤30%<br>V37Gy ≤50%	V42.75Gy >99%<br>Dmax ≤107%	33 months<br>(20-51)	60%	5%	5%	1%	44%	0%	17%	11%
Tree et al. [38]	SBRT	V37Gy ≤5.4 cm^3	V36Gy ≤1.3 cm^3<br>D50% ≤14.4Gy	V36.25Gy >95%	14 months	31.8%<br>(Grade II)	5.9%	NA	NA	14%<br>(Grade II)	0%	NA	NA
Vargas et al. [56]	Proton beam therapy	V39Gy <8 cm^3	V24Gy <35%<br>V33.6Gy <10%	V38Gy >95%	18 months	19.6%<br>(Grade II)	0%	17.5%<br>(Grade II)	0%	4.3%<br>(Grade II)	0%	7.5%<br>(Grade II)	0%
Madsen et al. (SHARP) [57]	SBRT	NA	V33.5 Gy ≤1.06 cm^3	V30.15Gy >99%	41 months<br>(21-60)	48.5%	2.5%	45%	0%	39%	0%	37.5%	0%
Hannan et al. [47]	SBRT	Dmax ≤105%<br>V18.3Gy ≤10 cm^3	Dmax ≤22.5Gy	V50Gy ≥95%<br>Dmax ≤105%	54 months<br>(1-90)	70.8%	0%	45.1%	5.5%	58.3%	2.2%	38.5%	6.6%
Katz et al. (2013) [48]	SBRT 35Gy	D50% ≤15.05Gy	D50% ≤14.35Gy	V35Gy ≥96%	72 months<br>(9-78)	76%	0%	8%	0%	80%	0%	6%	0%
	SBRT 36.25Gy	D50% ≤15.6Gy	D50% ≤15Gy	V36.25Gy ≥96%	60 months<br>(8-72)	79.5%	0%	17%	2%	77.9%	0%	10%	0%

NA, Non available ; GU, genitourinary toxicity ; GI, gastrointestinal toxicity ; Gy, Gray ; PTV, planned target volume ; SBRT, Stereotactic body radiation therapy ; EBRT, External beam radiation therapy ; ENI, elective nodal irradiation ; GI, grade 1 ; RT, radiotherapy
