# Supplementary material for: Spatial Distribution of Recurrence and Long-Term Toxicity Following Dose Escalation to the Dominant Intra-Prostatic Nodule for Intermediate–High-Risk Prostate Cancer: Insights from a Phase I/II Study
Source: Cancers (Basel). 2024 May 31;16(11):2097. doi: 10.3390/cancers16112097 (PMC11171188; doi:10.3390/cancers16112097)
Supplement: Supplementary file 1 [file cancers-16-02097-s001.zip › Suppl_Table_S2.pdf]

Supplementary Table S2 : Dosimetric parameters and Quality of Life assessments

Author	Dose bladder planned per protocol	Dose rectum planned per protocol	Dose PTV planned per protocol	Score	Urinary QoL					Bowel QoL				
					Baseline	M3	M6	M12	M24	Baseline	M3	M6	M12	M24
Cloitre et al. [22]	0.1 cm^3< 45Gy<br>Median dose < 20Gy	0.1 cm^3 < 41Gy<br>V25Gy < 20cm^3	V47.5Gy > 95%$_{DIN}$<br>V42.5Gy >99%$_{DIN}$	PR-25-EORTC	87%	83.7%	85.1%	77.8%	78.6%	98.4%	96%	94.7%	92.8%	94.4%
Musunru et al. [49]	V32Gy <10%<br>V35Gy <5%	V32Gy <10%<br>V35Gy <5%	V23.75Gy >99%<br>V33.25Gy >99%	EPIC	86.6%	87.1%	NA	86.7%	87.9%	94.1%	93.2%	NA	92.5%	92.4%
Elias et al. [55]	V32Gy ≤40%<br>D5cm^3 ≤34Gy	V28Gy ≤40%<br>V32Gy ≤33%<br>D5 cm^3 ≤33Gy	V33.25Gy ≥99%	EPIC	89%	NA	88%	86.5%	90%	93%	NA	93.5%	92%	92%
Vargas et al. [56]	V39Gy <8 cm^3	V24Gy <35%<br>V33.6Gy <10%	V36.1Gy ≥99.5%	EPIC	91.3%	87%	88.7%	85.9%	90.9%	96.4%	91.9%	87.6%	87.5%	89.2%
Chen et al. [51]	V37Gy <5 cm^3	V36Gy <1 cm^3	V36.25Gy ≥95%	AUA (N/35)	9	8.5	8	8.8	8	NA	NA	NA	NA	NA

PTV, planning target volume; QoL, quality of life; PR-25-EORTC, form PR25 (prostate module) of the European organization for research and treatment of cancer; EPIC, expander prostate cancer index composite ; DIN, dominant intraprostatic nodule ; AUA, American urological association ; NA, not applicable

PR25-EORTC : 0% meaning maximum perturbation and 100% meaning absence of symptoms/perturbation
EPIC : 0% meaning poorest urinary/bowel function and 100% meaning absence of symptoms/perturbation
AUA score : 0-7 meaning mild symptoms, 8-19 meaning moderate and ≥20 meaning severe symptoms
