# Supplementary figures and images for: Spatial Distribution of Recurrence and Long-Term Toxicity Following Dose Escalation to the Dominant Intra-Prostatic Nodule for Intermediate–High-Risk Prostate Cancer: Insights from a Phase I/II Study
Source: Cancers (Basel). 2024 May 31;16(11):2097. doi: 10.3390/cancers16112097 (PMC11171188; doi:10.3390/cancers16112097)

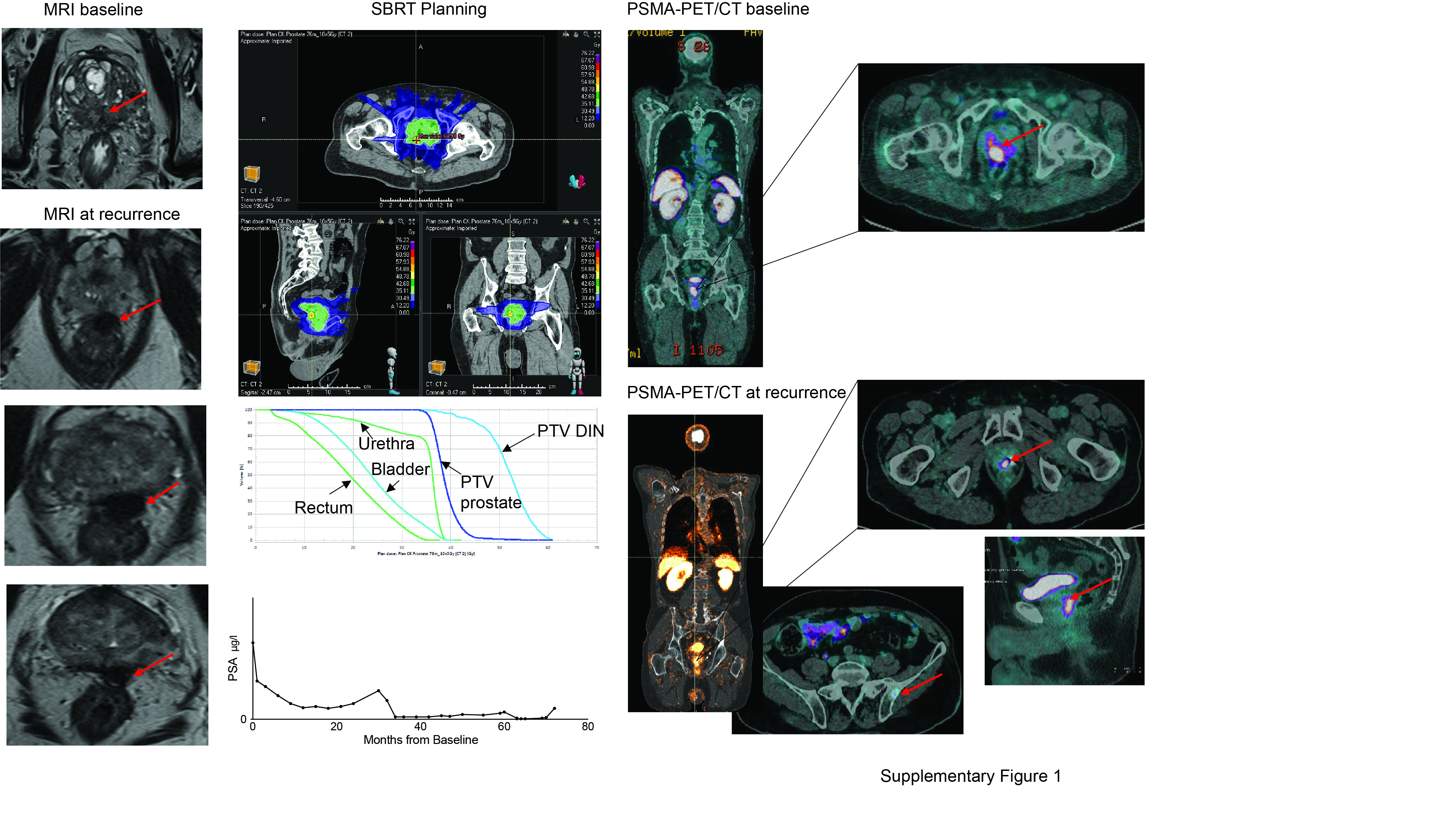

Supplement: Supplementary file 1 [file cancers-16-02097-s001.zip › Supp Figure S1.jpg]
